# Micronized Dispersible Ferric Pyrophosphate (MDFP) in Extruded Rice Desserts: High Iron Bioavailability and Good Sensory Acceptance by Children

**DOI:** 10.1111/1750-3841.70805

**Published:** 2026-01-11

**Authors:** Danielle Cristine Mota Ferreira, Thomás Valente de Oliveira, Maria Eliza Castro Moreira, Mônica Ribeiro Pirozi, Hércia Stampini Duarte Martino, Eduardo Basílio de Oliveira

**Affiliations:** ^1^ Department of Food Technology Federal University of Viçosa Viçosa MG Brazil; ^2^ Faculty of Chemical Engineering Federal University of Uberlândia Patos de Minas MG Brazil; ^3^ Department of Nutrition and Health Federal University of Viçosa Viçosa MG Brazil

## Abstract

Iron deficiency in children is a persistent global public health problem. This study evaluated the efficacy of an extruded rice matrix as a carrier for micronized dispersible ferric pyrophosphate (MDFP), aiming to increase iron bioavailability in traditional rice‐based desserts formulated with cow's milk (CM) or soy soluble protein extract (SSPE). Four experimental diets were administered to the rats: (i) fortified with CM and MDFP incorporated into extruded rice; (ii) fortified with SSPE and MDFP incorporated into extruded rice; (iii) control with ferrous sulfate (AIN‐93G); and (iv) a control with pure MDFP (AIN‐93G). Both fortified groups showed significantly higher recovery of iron nutritional status (*p* < 0.05), demonstrating that the different liquid ingredients did not compromise iron bioavailability. Gene expression analyses of key iron metabolism proteins (DMT‐1, DcytB, ferroportin, hephaestin, transferrin, and ferritin) confirmed that the extruded rice matrix increased the bioavailability of MDFP compared to the compound in its pure form (*p* < 0.05), possibly reducing aggregation and favoring direct interaction with intestinal absorption sites. Furthermore, the fortified formulations demonstrated satisfactory sensory acceptance among school‐ageRd children. These results indicate that incorporating MDFP into an extruded rice is an effective and technologically viable strategy for developing fortified rice desserts, with potential application in programs to combat iron deficiency anemia, specially in schoo‐laged children at poor regions worldwide.

**Practical Applications:**

Rice desserts fortified with micronized ferric pyrophosphate (MDFP) in an extruded rice matrix effectively improved iron availability without diminish sensory acceptance by children. Hence, the technology here reported offers a practical, low‐cost strategy for school feeding programs as well as for food industry to develop fortified products to fight against iron deficiency anemia.

## 1 Introduction

Iron is an essential micronutrient for the maintenance of cellular life, with homeostatic mechanisms that carefully regulate its utilization to prevent both deficiency and the harmful effects of excess. Despite significant advances in food science and nutrition, iron deficiency anemia remains a widespread micronutrient deficiency, affecting 24.3% of the global population, corresponding to 1.92 billion prevalent cases in 2021, and representing a major public health concern worldwide, disproportionately impacting women and children (Gardner et al. 2023). In 2019, according to the World Health Organization (https://www.who.int/), 39.8% of school‐aged children were affected by iron deficiency anemia (WHO 2025). The consequences of this deficiency in children extend beyond anemia, often resulting in reduced physical activity, increased fatigue, impaired concentration, and, in severe cases, compromised cognitive performance, growth, and immune function (Saengnipanthkul et al. 2025). Furthermore, iron deficiency may induce oxidative stress, potentially leading to cellular damage (Yadav et al. 2024). In light of this global scenario, the development of effective strategies to combat this health threat is of critical importance (Camaschella 2019).

To address iron nutritional deficiency, four main strategies are commonly recommended: (i) dietary diversification; (ii) supplementation; (iii) food fortification; and (iv) bio fortification (Shubham et al. 2020). Among these, food fortification offers advantages such as ease of acceptance and a favorable cost‐effectiveness ratio (Hemery et al. 2018). However, the bioavailability of iron in fortified foods is influenced by multiple factors, including the specific iron salt used, interactions with other minerals, and, critically, the food matrix itself (Diego Quintaes et al. 2017; Mantadakis et al. 2019; Parker et al. 2015). For instance, water‐soluble iron salts (e.g., ferrous sulfate) typically exhibit high bioavailability but may cause undesirable sensory changes in foods. In contrast, poorly water‐soluble salts (e.g., ferric pyrophosphate) may present lower bioavailability but are often more stable and induce fewer sensory alterations (Hurrell 2022). Reducing the particle size of ferric pyrophosphate has been shown to enhance its bioavailability, leading to the widespread use of MDFP in food fortification. The food matrix and its interactions with proteins and chelating agents also play a crucial role in determining iron bioavailability (Milman 2020).

The accessibility of iron‐fortified products to those in need remains a key limitation (Ge et al. 2025). In developing countries, providing low‐calorie iron‐fortified meals through school feeding programs can serve as a strategic approach to increase iron intake in children. In this context, the present study investigated formulations of rice‐based desserts, a traditional Latin American food made with rice and sucrose, fortified with dispersible MDFP. The study aimed not only to evaluate this strategy for combating iron deficiency in children but also to examine the impact of CM, SSPE, and, innovatively, an extruded rice matrix on iron absorption. Specifically, iron bioavailability and the gene expression of proteins involved in iron metabolism were assessed in Wistar rats fed these formulations, providing valuable insights into the underlying biological mechanisms and the potential of novel processing technologies to enhance food functionality. Additionally, the sensory acceptance of these formulations was evaluated by children, allowing us to verify their practical viability as a fortified product for them.

## 2 Materials and Methods

### 2.1 Formulations

The extruded rice grains, fortified with MDFP, were produced according to (Mishra et al. 2012) methodology, such as described in United States patent #5609896. Their production was performed using the URBANO company's (Brazil) facilities, kindly made available to us. These extruded grains had a shape and color remarkably similar to polished natural rice grains. Extruded grains were mixed with natural grains in the proportion (1:4), so their mix (from now on called simply “fortified rice”) contained about 54.5 mg of MDFP per kg.

Then, two different rice‐based dessert formulations were proposed, each with a different type of fluid protein ingredient: CM or SSPE, such that the dessert with fortified rice and CM was named R + CM, while the dessert with fortified rice and SSPE was named R + SSPE. The proportions of ingredients, centesimal composition, mineral contents (Ca, Fe and Zn), and caloric density for each formulation are summarized in Table 1.

**TABLE 1:** R + CM and R + SSPE formulations: centesimal composition, micronutrients (Ca, Fe, and Zn) contents and caloric density.

Composition	R + CM	R + SSPE
Moisture (g/100 g)	68.0 ± 2.30	66.6 ± 1.5
Carbohydrates (g/100 g)	25.91 ± 3.92	29,76 ± 1.48
Proteins (g/100 g)	3.17 ± 0.07	2.29 ± 0.05
Lipids (g/100 g)	1.24 ± 0.65	0.36 ± 0.04
Ashes (g/100 g)	0.75 ± 0.14	0.36 ± 0.04
Total dietary fiber (g/100 g)	0.001 ± 0.001	0.010 ± 0.001
Micronutrients	—	—
Iron (mg/100 g)	5.09 ± 0.08	5.93 ±1.04
Zinc (mg/100 g)	30.49 ± 0.95	29.99 ± 2.67
Calcium (mg/100 g)	91.53 ± 2.86	21.31 ± 0.96
Ca:Fe molar ratio	18:1	7:1
Zn:Fe molar ratio	6:1	5:1
Caloric density	127.48	131.44

*Note*: R + CM: rice and cow's milk; R + SSPE: rice and SSPE.

### 2.2 Centesimal Composition and Mineral Contents

All compositional and mineral analyses were performed on the final prepared and freeze‐dried formulations used in the AIN‐93G diets, ensuring that the reported values corresponded to the nutrients actually consumed by the animals.

Moisture content was evaluated by directly drying the samples in an oven (AOAC 1995). Total ash content was evaluated in a muffle at 550°C (AOAC 1995). The protein content was evaluated by the Kjeldahl method (AOAC 1995), assuming that all proteins present in the formulation were from rice (f_PTN_ = 5.95), CM (f_PTN_ = 6.38), or SSPE (f_PTN_ = 5.71) (FAO 2003). A multiplication factor for nitrogen content was calculated ponderously for each formulation: for R + CM, this factor was 6.31, whereas for R + SSPE it was 5.95. Lipid content measurent was performed using a Soxhlet fat extractor (AOAC 1995). The Fe, Zn, and Ca contents and total dietary fiber (F) were also all determined according to AOAC official protocols. Finally, total carbohydrate content was calculated by the difference between the total sample mass and the sum of the masses of other constituents (Table 1).

### 2.3 In Vivo Tests in Animal Models

The animal model study was conducted to evaluate the bioavailability of MDFP incorporated into an extruded rice matrix, comparing it with pure MDFP and ferrous sulfate.

**FIGURE 1:**
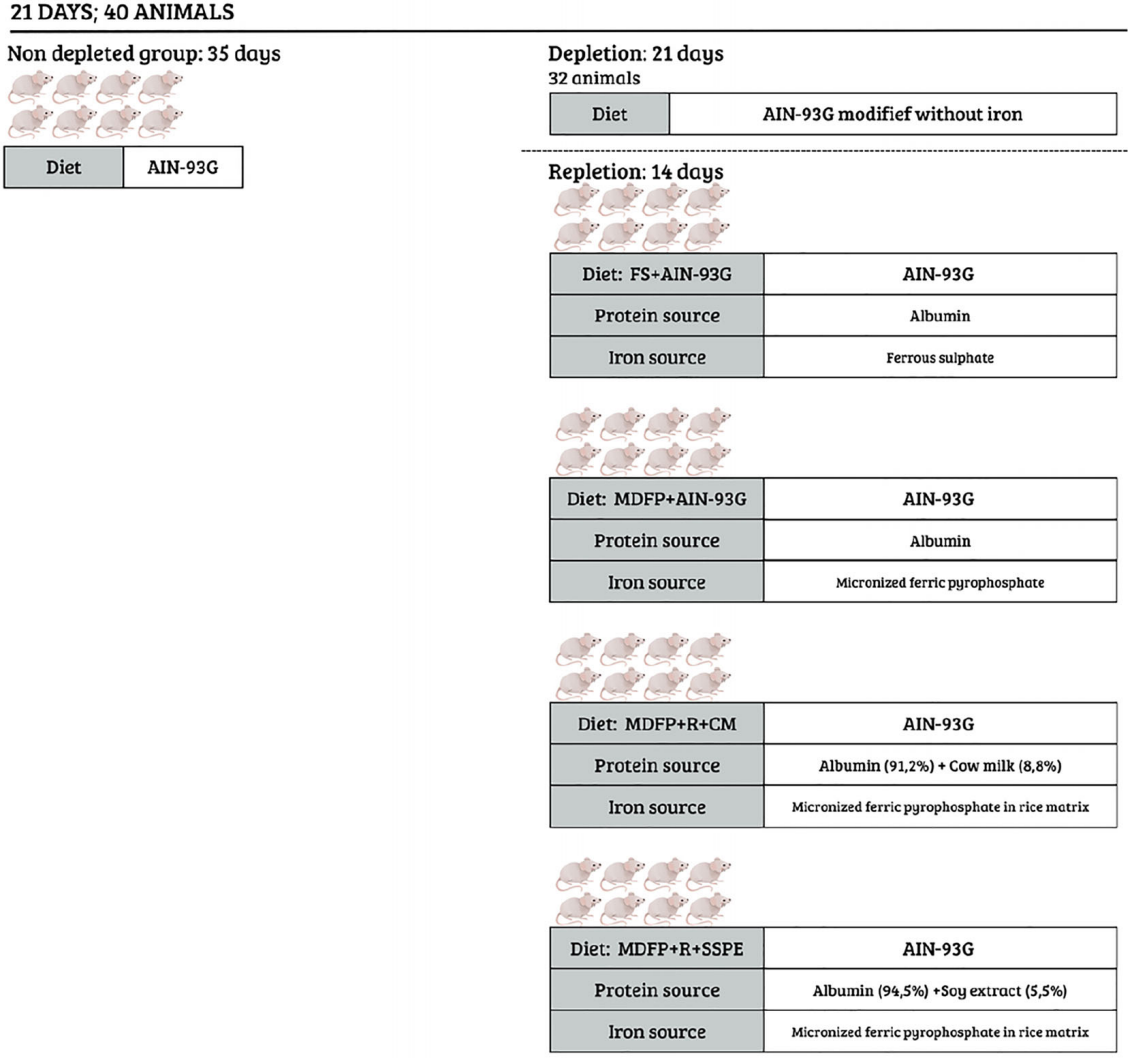
Experimental design. FS + AIN‐93G: diet with ferrous sulfate; MDFP + AIN‐93G: diet with MDFP; MDFP + R + CM: fortified dessert with rice + CM; MDFP + R + SSPE: fortified dessert with Rice + SSPE.

The hemoglobin depletion/repletion method was adopted for evaluating the bioavailability of iron (AOAC 2012). As shown in Figure 1, at 21 days of age, 40 male *albinus Wistar* rats (*Rattus norvegicus*) were placed in individual temperature‐controlled (21 ± 5°C) cages, with a photoperiod of 12 h. All procedures involving animals were approved by the Ethics Committee on Animal Use of the Federal University of Viçosa (CEUA/UFV, protocol no. 66/2015) and conducted in accordance with Brazilian guidelines for the care and use of laboratory animals.

Then, during the 21 days of the depletion phase, thirty‐two animals received an AIN‐93G diet modified by using mixtures of minerals without iron and deionized water ad libitum, for the induction of anemia (Reeves et al. 1993). Then, in the repletion phase (14 days), the rice‐based dessert formulation with CM (R + CM) or with SSPE (R + SSPE) was used as a source of iron in MDFP form. Two other different iron sources were used as controls: pure micronized dispersible ferric pyrophosphate (MDFP), to verify its bioavailability without the influence of the fortified rice matrix; and pure ferrous sulfate (FeSO4) (FS), to be used as a standard for iron bioavailability. Their amounts were calculated to provide 12 mg of iron per kg of diet in each treatment. The thirty‐two animals were placed into four groups (*n* = 8 in each group), which were fed differently: in MDFP form.

- MDFP + R + CM: group fed with a fortified diet containing extruded rice as source of MDFP and CM;
- MDFP + R + SSPE: group fed with a fortified diet containing extruded rice as source of MDFP and SSPE;
- FS + AIN‐93G: control group fed with an AIN‐93G diet containing pure ferrous sulfate (FeSO4) as an iron source;
- MDFP + AIN‐93G: control group fed with an AIN‐93G diet containing pure MDFP as an iron source.

A normal control group formed by eight animals received normal AIN‐93G diet (with iron) throughout these 35 days, without depletion phase.

The desserts were cooked, frozen, freeze‐dried, milled, and incorporated into the AIN‐93G diet, following the AOAC Official Method 971.30 (Rat Hemoglobin Repletion Test) (AOAC 2012), which requires a homogeneous and microbiologically stable diet for controlled bioavailability studies. Freeze‐drying was applied as a non‐thermal stabilization process to preserve the physicochemical integrity of the extruded rice matrix encapsulating the MDFP, ensuring uniform distribution and precise control of iron intake among animals.

The mineral content determination for the flour from R + CM and R + SSPE was performed according to the AOAC methods (AOAC 1995). The ingredients sugar, albumin, oil, microcrystalline cellulose, and corn starch had their proportions adjusted to ensure isocaloric diets (Table 2).

**TABLE 2:** Food and nutritional composition of diets with which rats were fed all along in vivo bioavailability and gene expression experiments.

Ingredient (per kg of diet)	Standard diet with iron (AIN‐93G)	Standard diet without iron	FS + AIN‐93G	MDFP + AIN‐93G	MDFP + R + CM	MDFP + R + SSPE
Albumin (g)	218.2	218.2	218.2	218.2	199.0	206.3
Dextrinized starch (g)	132.0	132.0	132.0	132.0	132.0	132.0
Sucrose (g)	100.0	100.0	100.0	100.0	45.1	24.4
Soybean oil (mL)	70.0	70.0	70.0	70.0	64.2	68.5
Microcrystalline cellulose (g)	50.0	50.0	50.0	50.0	49.4	49.6
Mineral mix with iron (g)	35.0	—	—	—	—	—
Mineral mix without iron (g)	—	35.0	35.0	35.0	35.0	35.0
Vitamin mix (g)	10.0	10.0	10.0	10.0	10.0	10.0
L‐cystine (g)	3.0	3.0	3.0	3.0	3.0	3.0
Choline bitartrate (g)	2.5	2.5	2.5	2.5	2.5	2.5
Corn starch (g)	379.3	379.3	379.2	379.2	310.8	330.2
Ferrous sulfate (mg)	—	—	80.13	—	—	—
Micronized ferric pyrophosphate (mg)	—	—	—	11.95	—	—
UR‐M (g)	—	—	—	—	149.0	—
UR‐S (g)	—	—	—	—	—	138.4
**Minerals**	**—**	**—**	**—**	**—**	**—**	**—**
Iron—Fe (mg/kg)	—	—	11.11	15.30	12.78	13.79
Calcium—Ca (mg/kg)	—	—	4635.0	4430.0	4795.0	4295.0
Ca:Fe molar ratio	—	—	581.32	403.45	522.80	433.99
**Caloric density (kcal/g)**	3.95	3.95	3.96	3.96	3.51	3.57

*Note*: FS + AIN‐93G: diet with ferrous sulfate; MDFP + AIN‐93G: diet with MDFP; MDFP + R + CM: fortified dessert with rice + cow's milk; MDFP+R+SSPE: fortified dessert with Rice + SSPE.

The animals received deionized water ad libitum and a controlled diet, approximately 15 g, for a period of 14 days. Food intake and weight gain were determined weekly by weighing, and the feed efficiency ratio was calculated by Equation 1, where TI refers to the total intake (g) and WI, to weight gain (g).

(1) $$FER=\frac{TI}{WI}$$

On the 36th day, after fasting for 12 h, the animals were anesthetized with isoflurane (Isoforine, Cristália) and euthanized by cardiac puncture. Blood and fragments of the liver, duodenum, and spleen were collected.

#### 2.3.1 Blood Tests and Iron Bioavailability

Serum hemoglobin was measured by the cyanide methemoglobin method, proposed by the AOAC (AOAC 2012), using a colorimetric kit for in vitro diagnosis (Bioclin). The volume of 10 µL of blood was pipetted and mixed with 2.5 mL of Drabkin's solution color reagent (containing potassium cyanide and hydrogen cyanide). The absorbance was analyzed in a UV‐visible Multiskan (thermo scientific), at a wavelength of 540 nm.

The iron bioavailability was calculated according to Hernández et al. (2003). The hemoglobin regeneration efficiency (HRE%) and the iron content in hemoglobin were calculated by Equation 2 and Equation 3, respectively. The iron content in hemoglobin was calculated assuming a total blood volume of 6.7% of the rat body weight and body iron content in hemoglobin of 0.335.

(2) $$HER=\frac{Fe_{end}Hb\left(mg\right)-Fe_{initial}Hb\left(mg\right)}{Fe_{consumed}\left(mg\right)\times 100}$$

(3) $$Fe_{\mathrm{Hb}}=\frac{\mathrm{Body}\_\mathrm{weig}\mathrm{ht}\left(g\right)\times \left[\mathrm{Hb}\right]\left(\frac{g}{L}\right)\times 0.335\times 6.7}{1000}$$

#### 2.3.2 Total mRNA Extraction From the Duodenal Mucosa and Liver

The liver and duodenum were macerated in liquid nitrogen under RNAse free conditions and the samples were aliquoted for total RNA extraction. Total RNA was extracted with TRIzol reagent (Invitrogen). After extraction, the RNA samples were treated with DNase (RQ1 RNAse‐free DNAse kit; Promega, USA). Total RNA was quantified at 260 nm in a spectrophotometer, and the degree of purity was quantified by the optical density ratio of 260/280 nm.

The isolated total mRNA was treated with DNAse (Invitrogen) and used for cDNA synthesis, using the M‐MLV Reverse Transcription kit (Invitrogen). Briefly, 2 µg of the mRNA extracted were added to 1 µL of 100 µM oligodT, and 1 µL of 10 µM dNTPs, diluted and incubated at 65°C for 5 min. Subsequently, the mixture was placed on ice and added 4 µL of 5x buffer FF, 2 µL of 0.1 M DTT, and 1 µL of RNAse Out. Then, 1 µL of MMLV reverse transcriptase (200 U/µL) was added and incubated in a water bath, at 37°C for 1 h. Finally, the cDNA was incubated at 70°C, for 10 min and quantified.

#### 2.3.3 Determination of Gene Expression of Proteins Involved in Iron Metabolism by Reverse Transcriptase Chain Reaction (RT‐qPCR)

The expression of mRNA levels for duodenal mucosa and liver proteins involved in iron metabolism was analyzed by reaction technique of real‐time polymerase chain (RT‐qPCR) (Dias et al. 2015). The markers SYBR green PCR master mix from Applied Biosystems (USA) and analyses were performed in a StepOne real‐time PCR system (thermo fisher scientific) equipment. The initial parameters used in the run were 20 s at 95°C, and then, 40 cycles with 30 s at 95°C for denaturation, 30 s of annealing at 60°C, and 1 min elongation at 72°C, followed by melting curve analysis. Sense and antisense primer sequences (GenOne Biotechnologies, Rio de Janeiro, Brazil) were used to amplify protein divalent metal carrier (DMT‐1), duodenal cytochrome b (DcytB), ferroportin, hephaestin (Sigma‐Aldrich, Missouri, MO, USA, and the duodenum and liver proteins: ferritin and transferrin (GenOne Biotechnologies, Rio de Janeiro, Brazil). The relative expression levels of mRNA were normalized by the endogenous control glyceraldehyde 3‐phosphate dehydrogenase (GAPDH) for rats. All steps were performed under open RNAse conditions.

### 2.4 In Vivo Trials With human

The study with children was conducted to evaluate the sensory acceptance of fortified rice desserts. All procedures were reviewed and approved by the Research Ethics Committee of the Federal University of Viçosa (approval no. 1.328.871, date of approval: November 19, 2015), and informed consent was obtained from parents or legal guardians before the participation of children.

For this evaluation, the two rice formulations developed were incorporated into the traditional rice pudding recipe, preserving their original culinary characteristics. After preliminary testing, using a standard recipe, the composition of each formulation was established as described in Table 3. The sucrose ratio was adjusted to obtain a fluid mixture (“CM + sucrose” or “6% SSPE + sucrose solution”) with the same total soluble solids content.

**TABLE 3:** Defined composition for each formulation of the rice pudding recipe.

Ingredients (%)	Formulation	
	CM	SSPE
Common rice	8.4	8.4
Extruded rice	2.6	2.6
Cow's milk	78.0	—
Solution of SSPE (6%)	—	71.6
Other ingredients	—	—
Sucrose	10.5	17.0
Vanilla flavor	0.50	0.50
Salt	0.06	0.06
Water for cooking*	9x	9x

Abbreviations: CM: Cow's milk; SSPE: soluble soybean extract.

In the fortified formulations containing CM and soluble soybean extract (SSPE), the total amount of rice consisted of a blend of common rice (CR–75%) and extruded rice (ER–25%). This proportion ensured that, after processing, the desserts had an average iron content of 4.9 mg per 90 g serving, exceeding 30% of the dietary reference intake (DRI)—the minimum required by ANVISA (1998) for a product to be classified as fortified.

#### 2.4.1 Production Process

**FIGURE 2:**
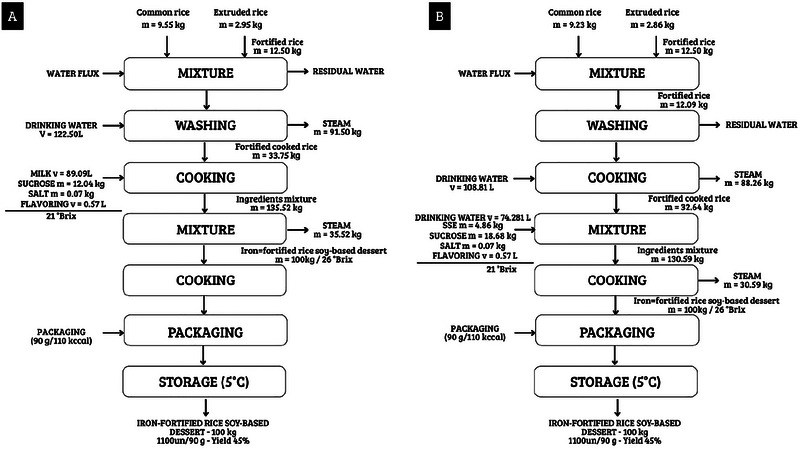
Schematic representation of the rice pudding production process, including mass balance of 100 kg of desserts containing (A) CM; and (B) SSPE. Residual water: dirty water after washing.

The production process, including a mass balance, considering 100 kg of fortified formulations, is shown in Figure 2A (CM) and Figure 2B (SSPE).

#### 2.4.2 Consumer Sensory Acceptance Testing

**FIGURE 3:**
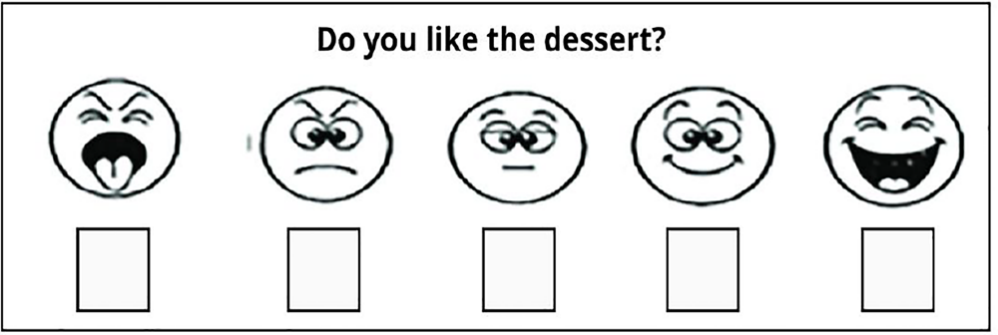
Sensory evaluation questionnaire.

Sensory evaluations were carried out with 250 children of both sexes in school age (5–9 years) and pre‐school age (3–4 years). The formulations were prepared and distributed according to the specifications of the Brazilian National School Feeding Programme (PNAE) (BRASIL 2014). Portions of 90 g were placed into cups of 100 mL and offered to each child individually, along with an acceptance questionnaire (Figure 3).

This questionnaire contained five illustrations of facial expressions related to their opinion about the product consumed, simulating a hedonic scale with five points. The score five corresponds to “very appreciate”, the four to “appreciate”, the three to “indifferent”, the two to “dislike” and the scores one to “very dislike” For each formulation, a mean from the scores assigned by the children was calculated. Two acceptance classifications were assumed: “good acceptance” for average scores >3, and “bad acceptance” for average scores ≤3.

Children under five years old were unable to answer the questionnaire. Therefore, the sensory acceptance was also assessed through the test of “intake remainder”, with modifications (Cecane 2017). In this test, the acceptability of the respective preparations was evaluated by the acceptability index (AI), which relates the amount consumed to the amount initially distributed according, to equation (Equation 1), in which “TMS” is the total mass served and “R” mass that was not ingested.

Two acceptance classifications were assumed: “good acceptance” for IA >70%, and “bad acceptance” for IA ≤70% (Teixeira et al. 1987).

(4) $$AI=\frac{TMS\;-\;R}{TMS}\;$$

### 2.5 Statistical Analyses

All experiments followed a completely randomized design. In the animal studies, each rat (*n* = 8 per group) was considered an independent experimental unit. For the chemical composition analyses, measurements were performed in triplicate using independently prepared samples. In the sensory evaluation, each child's assessment was treated as one replicate.

Data were expressed as mean ± standard deviation (SD). The assumptions of normality and homogeneity of variance were verified using the Shapiro–Wilk and Levene's tests, respectively. When assumptions were met, data were analyzed by one‐way ANOVA at *α* = 0.05. Post‐hoc comparisons among treatment means were performed using Tukey's test. Orthogonal contrasts were applied when appropriate to further explore treatment effects.

All statistical analyses were performed using SAS software (version 9.0; SAS Institute, Cary, NC, USA).

## 3 Results and Discussion

### 3.1 In Vivo Tests in Animal Models

#### 3.1.1 Iron Viability

The iron content of the two fortified desserts prepared with cow milk (R + CM) and with a soy soluble protein extract (R+SSPE) did not differ significantly (*p* > 0.05), averaging 5.5 mg of iron per 100 g of dessert. This amount provides approximately 30% of the dietary reference intake (DRI) for children aged 3 to 10 years (5.8 to 8.9 mg/day), thus qualifying both formulations as iron‐fortified foods (Hu et al. 2024; Hurrell 2022). However, due to the presence of milk in the R + CM formulation, this dessert exhibited significantly higher levels (*p* < 0.05) of calcium, protein, and lipids compared to the R + SSPE formulation (Table 1).

The higher sucrose content in the SSPE formulation was intentionally adjusted to balance sweetness and texture with the milk‐based desserts (which contain lactose), aiming at ensuring similar acceptability in both formulations. This indicate that a 90 g portion of the dessert, consistent with the serving size recommended by the Brazilian National School Feeding Programme (BRASIL 2014), provides approximately 9 to 10 g of total sugars, corresponding to less than 10% of daily energy intake according to the WHO (2025) recommendations for free sugar consumption in children.

**FIGURE 4:**
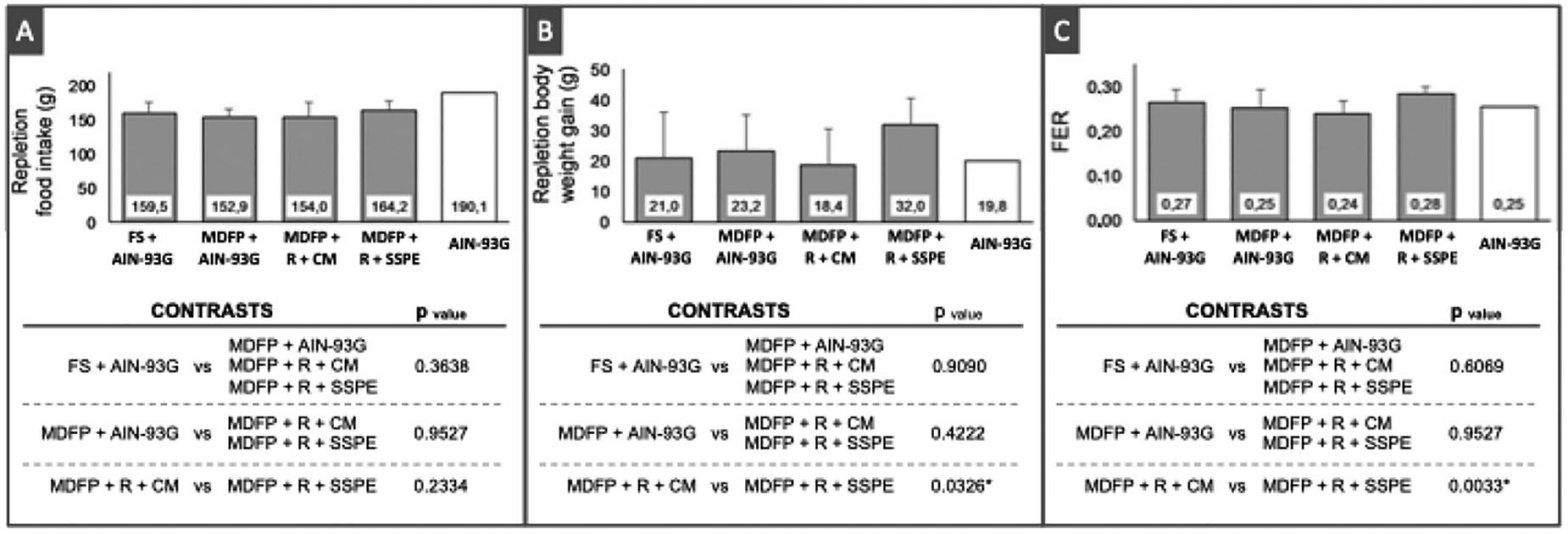
Total intake (A), body weight gain (B) and food efficiency ratio (FER) (C) of Wistar rats (*n* = 8). FS + AIN‐93G: diet with ferrous sulfate; MDFP + AIN‐93G: diet with MDFP; MDFP + R + CM: fortified dessert with rice + CM; MDFP + R + SSPE: fortified dessert with rice + SSPE. * Significant difference among contrasts groups (*p* < 0.05).

The total consumption during the repletion period was not different among the groups (*p* > 0.05) (Figure 4a). Animals fed with the MDFP + R + SSPE diet presented higher weight gain (*p* < 0.05) than those fed the MDFP + R + CM diet (Figure 4b–c). This finding supports recent studies suggesting that protein‐based systems, such as those derived from soy, may have a favorable impact on growth in iron‐deficiency models, regardless of the type of iron supplement used, by not impairing the recovery of nutritional status (Hu et al. 2024).

Since the diets had no significant difference in terms of caloric density, a hypothesis to explain this higher FER observed for rats fed the MDFP + R + SSPE diet is the fact that proteins with higher lysine content, such as soy proteins, are well‐known to possess a better capacity to convert the feed intake into body mass. In fact, soy proteins have roughly 8–9% of lysine, while milk whey proteins have 6–7% (Cheng et al. 2017).

**FIGURE 5:**
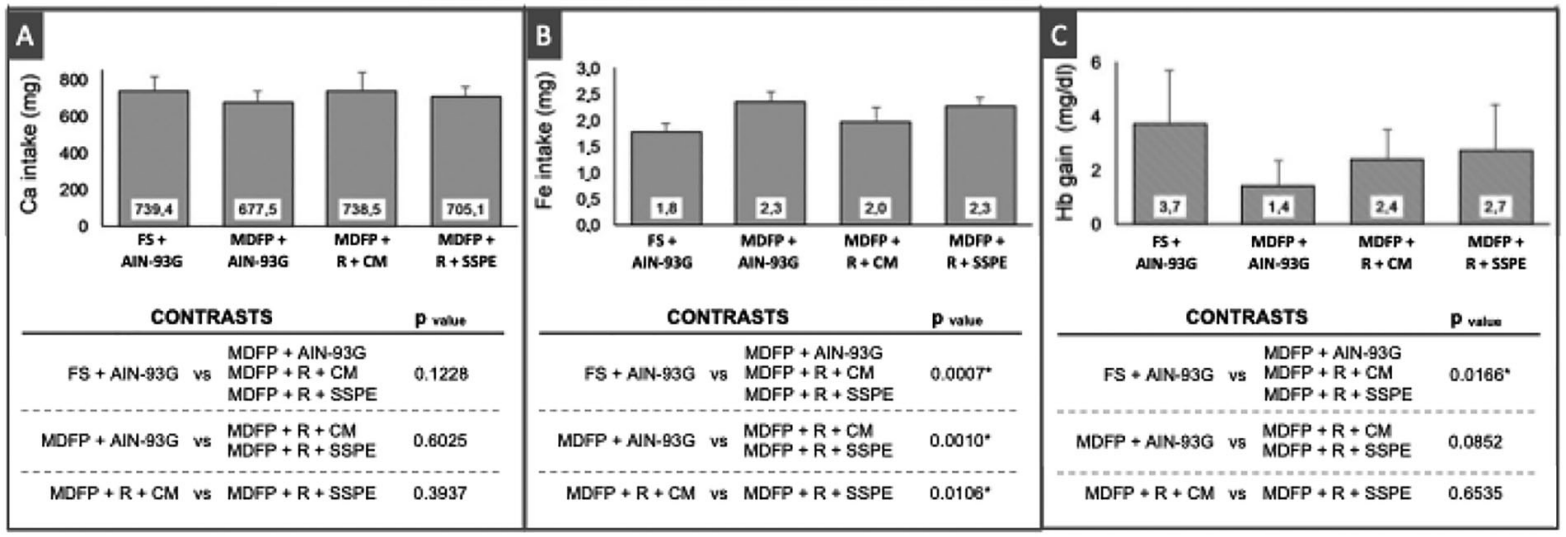
Ca intake (A), Fe intake (B) and Hb gain (C) of Wistar rats (*n* = 8). S FS + AIN‐93G: Diet with ferrous sulfate; MDFP + AIN‐93G: Diet with MDFP; MDFP + R + CM: fortified dessert with rice + CM; MDFP + R + SSPE: fortified dessert with rice + SSPE.

Although the MDFP + R + CM diet presented a higher concentration of calcium, the calcium intake was not significantly different among all animal groups (*p* > 0.05) (Figure 5a).

On the other hand, the control group with ferrous sulfate (FS + AIN‐93G) presented the lowest iron intake among all groups (*p* < 0.05), while the MDFP control group (MDFP + AIN‐93G) showed the highest iron intake (*p* < 0.05) (Figure 5b). However, despite these differences in total iron intake, the four experimental groups recovered their nutritional iron status after the repletion period (Figure 5b). It is also observed that the Ca:Fe molar ratio (from 403 to 582 mol Ca/mol Fe) showed no correlation with the bioavailability of the iron from the studied diets. In agreement with these results, it has been reported that milk and its components do not seem to negatively affect Fe absorption in anemic rats. In this in vivo study, Sachdeva et al. (2015) observed that the iron bioavailability was more influenced by the iron nutritional status than the total iron intake during the repletion. Another study, with the participation of 54 healthy non pregnant women, showed that nonheme iron absorption was significantly reduced only when the iron compound was in taken simultaneously with more than 800 mg of Ca, in calcium chloride form (Gaitán et al. 2011). They also noted that calcium doses >1000 mg diminished the 5 mg nonheme iron absorption by an average of 49.6% and a calcium dose of 800 mg diminished absorption of 5 mg heme iron by 37.7% (Gaitán et al. 2011)^.^ Blanco‐Rojo and Vaquero (2019) pointed out that this iron bioavailability decrease is caused by the interactions between calcium and other divalent with DMT‐1, an iron transport protein into the enterocytes. Therefore, the calcium contents in rice‐based dessert with CM (MDFP + R + CM) may not be enough to affect iron absorption, whereas the adequate portion of this dessert for one child is about 100 g, which allows an iron intake of around 5.09 mg, on average, so that 2.7 mg is bioavailable, and a calcium intake of 91.53 mg, on average (Blanco‐Rojo and Vaquero 2019).

However, in the context of a typical portion of the rice‐based dessert with CM, (MDFP + R + CM), which provides an average iron intake of around 5.09 mg (with 2.7 mg bioavailable) and a calcium intake of 91.53 mg per 100 g, the calcium content may not be sufficient to negatively impact iron absorption. This observation is further supported by recent findings from Hu et al. (2024), who reported that iron supplementation in soy protein systems had similar beneficial effects on body iron status and gene expression of key iron absorption proteins compared to whey protein systems, implying that protein source (and associated calcium) did not adversely affect iron absorption in suckling rats. This suggests that the protein matrix itself might play a more dominant role or that the calcium levels present did not reach an inhibitory threshold.

It is important to note that, despite the lower iron consumption (Figure 5c), the ferrous sulfate control group (FS + AIN‐93G) showed better hemoglobin recovery (*p* > 0.05) than the MDFP control group (MDFP + AIN‐93G), which suggests better iron bioavailability from FeSO_4_ than that from MDFP, as already mentioned by Cercamondi et al. (2016). However, rats fed the diet containing MDFP inserted within the matrix made of extruded rice grain (MDFP + R + CM and MDFP + R + SSPE groups) did not differ significantly from the group that ingested FeSO_4_ (*p* > 0.05), regarding the recovery of their iron nutritional status. This result demonstrates that the matrix of extruded rice positively affects the bioavailability of iron when using MDFP. Similar results were reported by Moretti et al. (2006), in which the MDFP presented better bioavailability inside the rice grain than pure MDFP, with relative bioavailability values equal to 24% and 15%, respectively. It demonstrates that MDFP bioavailability varied markedly according to the food matrix.

**FIGURE 6:**
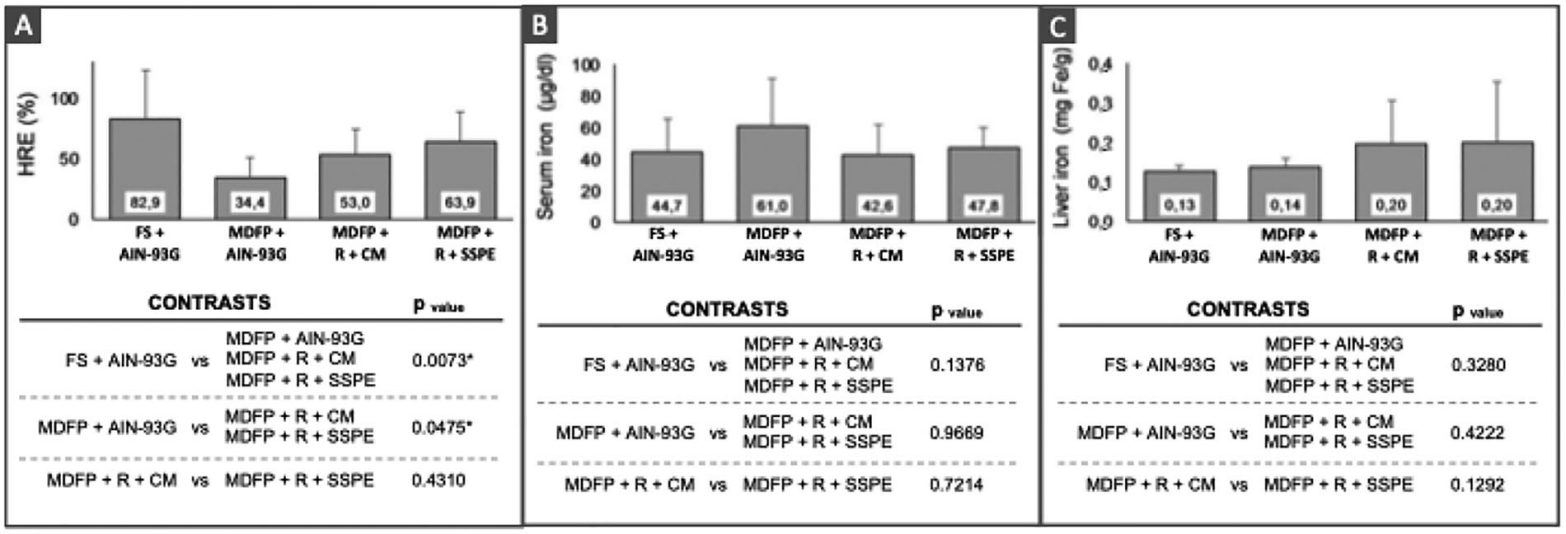
Hemoglobin regeneration efficiency—HRE (A), serum iron (B) and liver iron (C) of Wistar rats (*n* = 8). S FS + AIN‐93G: diet with ferrous sulfate; MDFP + AIN‐93G: diet with MDFP; MDFP + R + CM: fortified dessert with rice + CM; MDFP + R + SSPE: fortified dessert with Rice + SSPE.

Regarding the hemoglobin regeneration efficiency (HRE), the FS + AIN‐93G control group presented better HRE (*p* < 0.05) than MDFP + AIN‐93G control group and groups fed diets formulated with fortified desserts (MDFP + R + CM and MDFP + R + SSPE groups) (Figure 6a).

Such results are corroborated by different studies on iron‐fortified products, which also concluded that FeSO_4_ has higher bioavailability than MDFP (Cercamondi et al. 2016; Moretti et al. 2006). This variation could be due to differences in their uptake into the intestinal cells, since, before absorption, Fe^3+^ must be reduced to Fe^2+^ by a reducing agent, duodenal cytochrome b (Dcytb), or other reducing components on the brush border membrane, to be transported by the apical divalent metal transport protein 1 (DMT1) to the enterocyte (Caballero Valcárcel et al. 2019b; Cercamondi et al. 2016; Cheng et al. 2017; Latunde‐Dada et al. 2002). On the other hand, some researchers obtained contradictory results, wherein the MDFP presented greater bioavailability of iron than FeSO_4_, suggesting that decreasing particle size in MDFP has been an effective strategy to improve iron solubility at low pH (Diego Quintaes et al. 2017).

**FIGURE 7:**
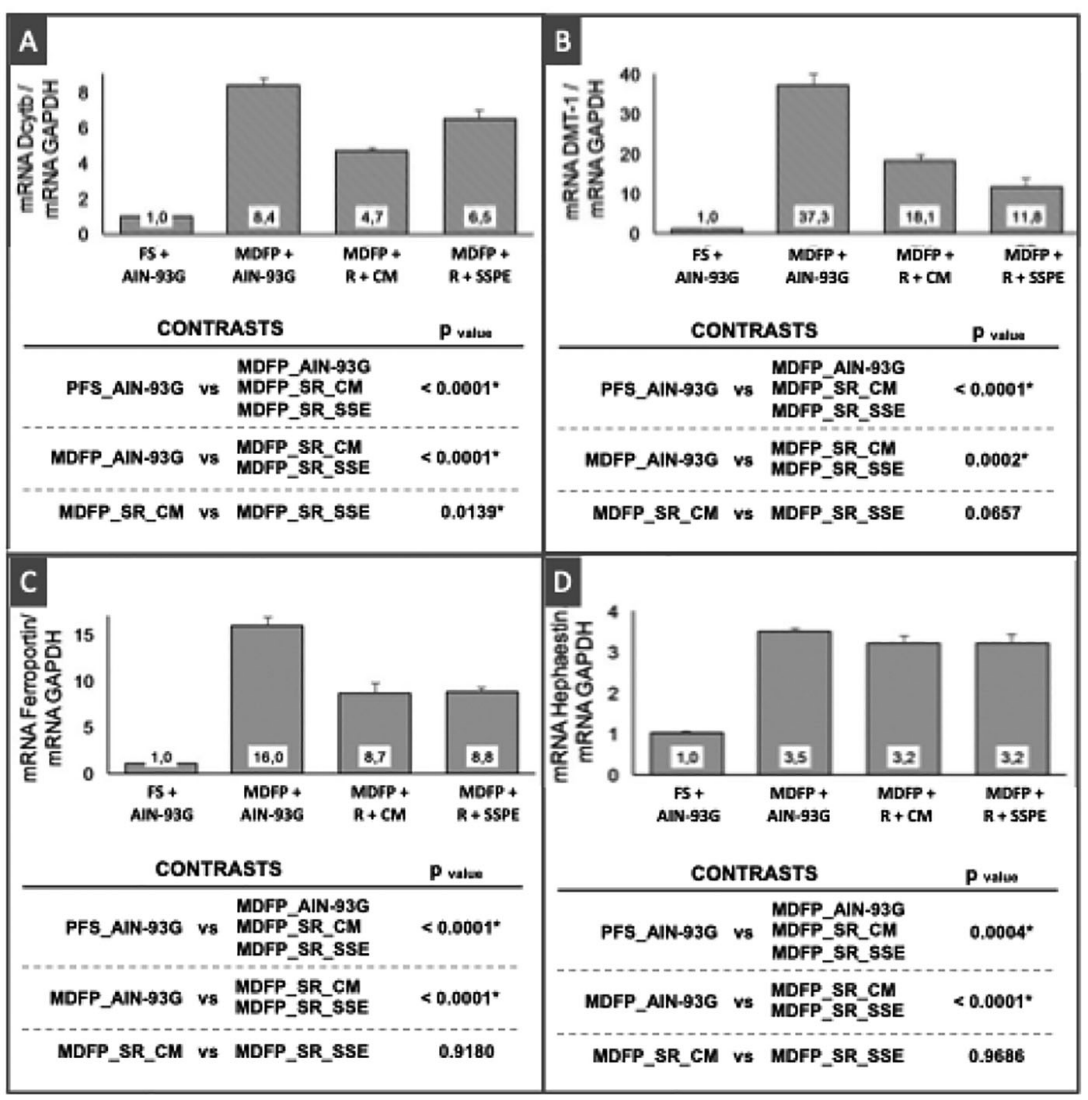
Effect of the ingestion of different iron sources on the gene expression of proteins in duodenal tissue. RT‐PCR analysis. DcytB (A), DMT‐1 (B), ferroportin (C), hephaestin (D). FS + AIN‐93G: diet with ferrous sulfate; MDFP + AIN‐93G: diet with MDFP; MDFP + R + CM: fortified dessert with rice + CM; MDFP+R+SSPE: fortified dessert with Rice + SSPE.

The control group with pure MDFP (MDFP + AIN‐93G) consistently exhibited lower HRE (*p* < 0.05) compared to groups where MDFP was integrated into the extruded rice matrix (MDFP + R + CM and MDFP + R + SSPE groups), with no significant differences observed between these two rice‐matrix groups (Figure 6a). This indicates a clear enhancement of MDFP bioavailability by the extruded rice matrix. Despite these HRE differences, serum and liver iron content did not differ significantly (*p* > 0.05) among the four experimental groups (Figure 4b–c), suggesting that while the initial absorption rate might differ, the overall iron repletion was achieved. This behavior can be attributed to some factors: firstly, it is likely that the extruded rice matrix protected Fe cations all along its passage through the gastrointestinal tract, almost as a microcapsule, limiting iron immobilization by other diet compounds and increasing its accessibility for uptake (Bryszewska 2019; Jati Kusuma and Ermamilia 2018). This strategy, where a food matrix or protein‐based system acts as a protective delivery vehicle for iron fortificants with low solubility, aligns with recent advances in food bioscience (Fan et al. 2022). This hypothesis is further supported by the results of DcytB gene expression (Figure 7a) in which rats of the MDFP + AIN‐93G control group showed higher gene expression than those constituting the MDFP + R + CM and MDFP + R + SSPE groups (*p* < 0.05). Therefore, it is reasonable to believe that, in animals fed with of pure MDFP (MDFP + AIN‐93G), a higher proportion of free iron (Fe^3+^) has reached the enterocyte brush border, inducing the DcytB expression. Another crucial aspect lies in the production process, as extrusion into the rice grain, as demonstrated by Hackl et al. (2017) and Moretti et al. (2006), can effectively fix the MDFP particles and minimize their potential aggregation, thereby enhancing their bioavailability. The cooking process to produce the desserts also affects the MDFP behavior, which agrees with Moretti et al. (2006), who found that heat sterilization of an infant formula containing ferric pyrophosphate increased its bioavailability by 50 %. Sodium chloride may also have improved the solubility of MDFP in the dessert formulation (Table 1), which suggests that iron absorption could be further improved, as observed by Cercamondi et al. (2016).

Similarly, the analysis of DMT‐1, which plays a crucial role in transporting Fe^2+^ into enterocytes, demonstrated that its gene expression was significantly more pronounced in the MDFP + AIN‐93G control group (*p* < 0.05) than in groups fed diets containing MDFP entrapped within the extruded rice (MDFP + R + CM and MDFP +R + SSPE groups) (Figure 7b) (Fidler et al. 2004).

This phenomenon is particularly insightful given the dynamic nature of iron homeostasis. Iron deficiency itself has been consistently reported to up regulate iron absorption capacity in mammals (Buffler et al. 2015). Thus, the higher expression of DcytB and DMT‐1 observed for the MDFP + AIN‐93G control group may be considered as a physiological response in these animals to improve their iron absorptive capacity, induced by the lower bioavailability of this mineral (*p* < 0.05), when compared to groups that fed with MDFP inside extruded rice. This aligns with findings from Valcárcel et al. (2022), who observed variations in DMT‐1 expression depending on the iron source and discussed the complex, dynamic regulation of iron transporters at the cellular level. Their work further suggests that less soluble iron forms, like MDFP, might be transported by alternative pathways, such as endocytosis, which could influence the observed gene expression patterns independent of a direct DMT‐1 up regulation.

Another crucial aspect lies in the production process: extrusion into the rice grain, as demonstrated by Hackl et al. (2017) and Moretti et al. (2006), can effectively fix the MDFP particles and minimize their potential aggregation, thereby enhancing their bioavailability. The cooking process to produce the desserts also affects MDFP behavior, which agrees with Moretti et al. (2006), who found that heat sterilization of an infant formula containing ferric pyrophosphate increased its bioavailability by 50%. Furthermore, sodium chloride may also have improved the solubility of MDFP in the dessert formulation (Table 1), which suggests that iron absorption could be further enhanced.

When the nutritional iron status of the animals decreased, increased gene expression was observed for both ferroportin (protein that transports Fe^2+^ from enterocytes to bloodstream) and hephaestin (protein directly involved in the conversion of Fe^2+^ into Fe^3+^, so that it can be incorporated into transferrin in the bloodstream) (Figure 7c–d). It suggests that the increased iron absorptive capacity observed in the MDFP + AIN‐93G control group can also be correlated to an increased gene expression of ferroportin and hephaestin. This is supported by the dynamic balance found in the contents of iron in the serum and liver of the animals (*p* > 0.05) among the groups controlled by the increased or decreased ferroportin and hephaestin gene expression (Figure 7c–d). Moretti et al. (2006) pointed out that the increased iron absorption after decreased iron stores is a central mechanism of iron homeostasis in humans. They also revealed that this adaptive up‐regulation of iron absorption is more effective for ferrous sulfate.

**FIGURE 8:**
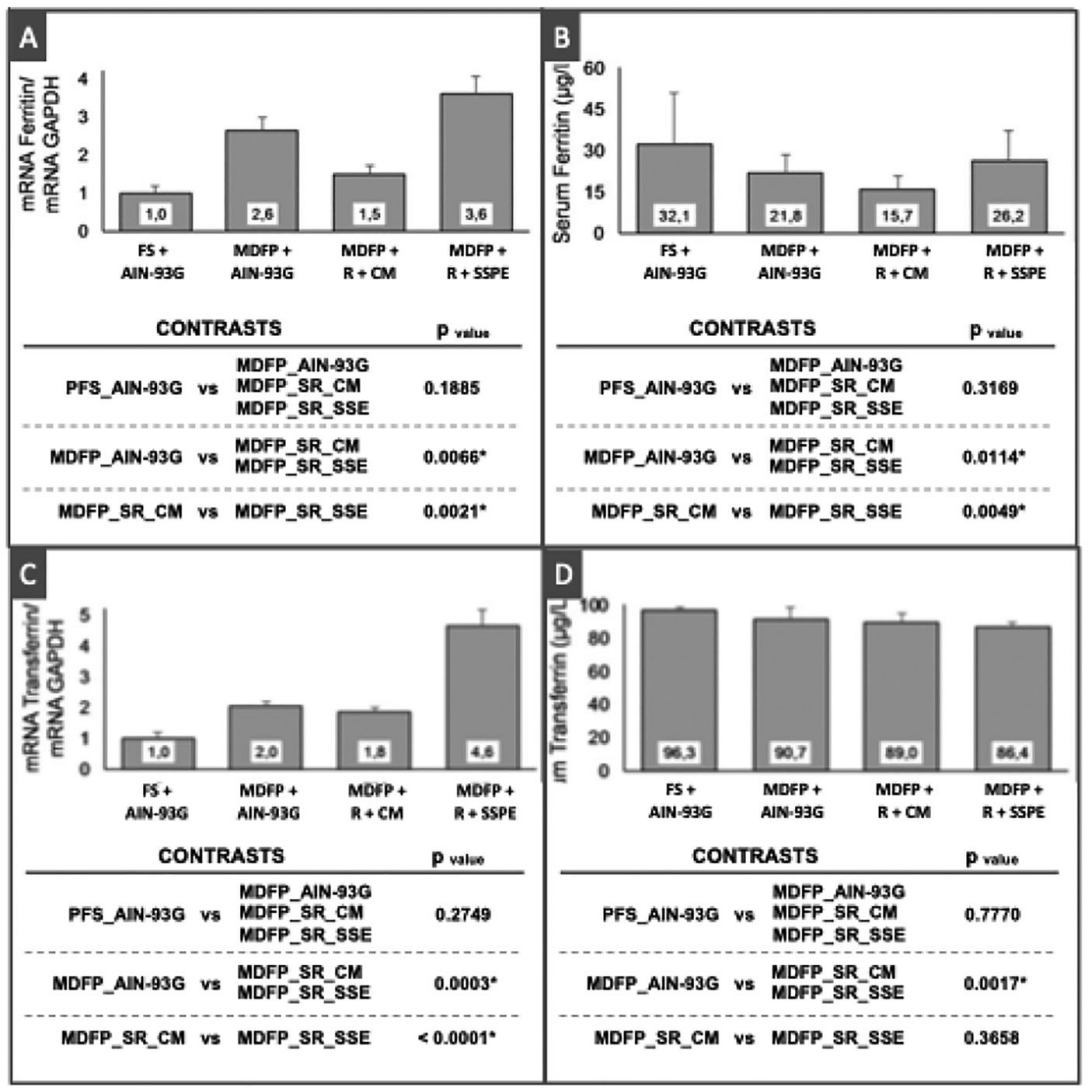
Effect of different iron sources intake on the gene expression of proteins in liver tissue and serum content of these proteins. RT‐PCR Analysis. Ferritin expression (A), serum ferritin content (B), transferrin expression (C), serum transferrin content (D). FS + AIN‐93G: diet with ferrous sulfate; MDFP + AIN‐93G: diet with MDFP; MDFP + R + CM: fortified dessert with rice + CM; MDFP + R + SSPE: fortified dessert with rice + SSPE.

It is also important to consider that decreased iron release into the bloodstream can reduce the expression of genes coding for the proteins involved in the storage and transport of the iron absorbed, such as ferritin and transferrin (Buffler et al. 2015). In the present study, both the gene expression of ferritin and serum ferritin content were higher in animals fed diets containing MDFP + R + SSPE, as shown in Figure 8a–b.

Although no statistical difference was detected between MDFP + R + CM and MDFP + R + SSPE groups (*p* > 0.05), the MDFP + R + SSPE group presented %HRE about 20% higher than MDFP + R + CM group, suggesting that the increased serum ferritin levels in animals fed with diets with SSPE can be correlated to their improved nutritional iron status, compared to those fed with diets with CM. Nonetheless, it is worth mentioning that this improvement could not be significantly expressed in %HRE (*p* > 0.05), due to the short repletion period.

Finally, the expression of transferrin (protein whose function is to transport iron from the bloodstream to the body tissues) was significantly higher for the MDFP + R + SSPE group, compared to the three other groups (*p* > 0.05) (Figure 8c–d). Such result is probably associated with the higher weight gain of the animals fed diet with rice‐based dessert containing SSPE (Figure 1b), as they presented higher FER (*p* > 0.05) at the end of the repletion phase. This condition may be desirable for individuals at a high tissue production stage, such as children and pregnant women, for the promotion of proper transport of Fe through the bloodstream and from the bloodstream to the tissues under development.

In summary, the fortified desserts formulated with MDFP entrapped within extruded rice matrices presented good relative bioavailability and the rice matrix improved MDFP bioavailability. Notably, the extruded rice grain matrix improved MDFP iron bioavailability, possibly due to Fe cation protection throughout the gastrointestinal tract of the rats, the extrusion and cooking production processes that minimize the potential aggregation of MDFP, and the addition of sodium salt (NaCl) in the dessert formulations, which improved MDFP solubility. This aligns with findings by Moretti et al. (2006), which demonstrated that the relative bioavailability of ferric pyrophosphate varied markedly with the food matrix and was higher when extruded into artificial rice grains compared to when added directly to an unprocessed rice meal. Since the calcium content in the rice‐based dessert formulated with CM (MDFP + R + CM) was not sufficient to reduce iron absorption, we believe that the product developed in this study (inexpensive and easy to produce) has great potential to be served as school meal to minimize iron deficiency in children. Moreover, we indicate MDFP to be used as a fortifying agent in other food matrices, since it does not negatively affect the sensory attributes, which gives it good relative bioavailability.

### 3.2 In Vivo Trials With Human

**FIGURE 9:**
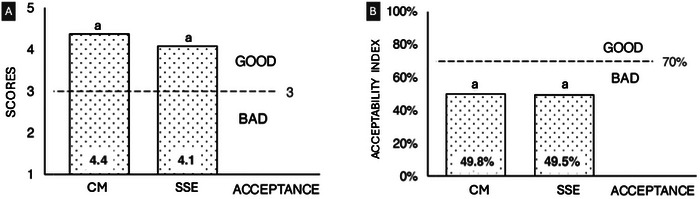
Results of sensory acceptance testing by acceptance questionnaire (A) and intake remainder (B). means with different letters represent statistical difference (*p* < 0.05) by Tukey test. Rice desserts containing (A) CM (CM) and (B) SSPE.

The sensory evaluation by children revealed a significant contrast between the methods applied. Using the five‐point hedonic questionnaire (Figure 9A), all formulations, both those with CM and those with SSPE achieved averages >3, classifying them as “good acceptance” regardless of the age group evaluated. This result suggests good initial acceptance, likely associated with the school context and the playful format of the scale, which may minimize explicit negative responses.

The leftover consumption test (Figure 9B), on the other hand, showed greater sensitivity in identifying differences in preference. In this test, both puddings (CM and SSPE) presented acceptability indices (AI) below 70%, being classified as “low acceptance” according to this criterion, with values close to 49.8% and 49.5%, respectively. Although there was no statistical difference, the method showed an overall actual acceptance, measured by actual consumption, was lower than the perception stated by the questionnaire's responses.

**FIGURE 10:**
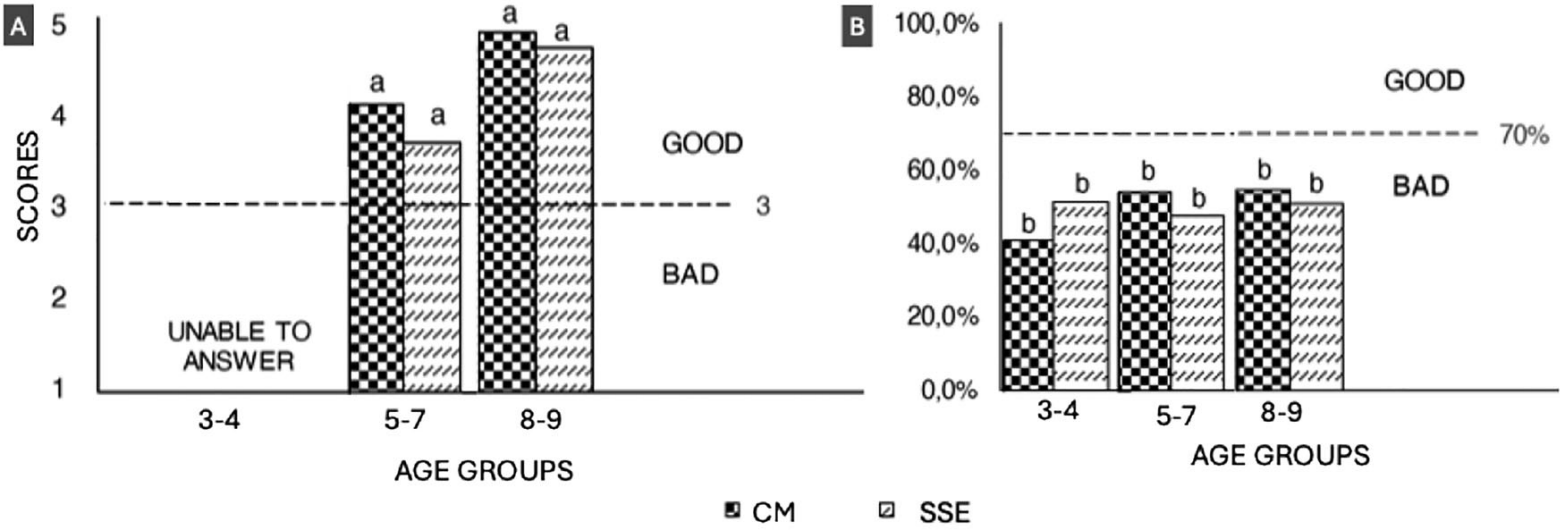
Sensory acceptance testing by age groups by acceptance questionnaire (A) and intake remainder (B). Means with different letters represent statistical difference (*p* < 0.05) by Tukey test. Rice desserts containing: (A) CM; and (B) SSPE.

Analysis by age group (Figures 10A and 10B) confirmed the absence of significant differences in the hedonic questionnaire between the 3–4, 5–7, and 8–9 age groups, suggesting that sensory perception was homogeneous across age groups. However, in the leftover consumption test, all age groups presented an AI <70% (Figure 10B), reinforcing that practical adherence to the product can be limited, regardless of age.

These differences between methods may be associated with children's greater familiarity with the taste and aroma of CM in sweet preparations, the contribution of the higher lipid content to a creamier texture and mouthfeel sensation, and possible residual notes characteristic of soy, which historically reduce children's acceptance (Bachmann 2001). Recent studies confirm this trend, Hasan et al. (2024) observed that dairy‐free frozen desserts formulated with plant‐based proteins, although less acceptable than the dairy‐based version, remained at acceptable levels, especially when soy and pea‐based.

Texture and volatile profile modulation is another critical factor. McCarron et al. (2024) showed that even small additions of plant‐based protein (pea or potato) to oat beverages significantly altered the sensory profile and generated undesirable volatile compounds, affecting acceptance. This observation reinforces the need to optimize SSPE formulations, either by reducing volatile compounds associated with soy or by increasing creaminess. Jaeger et al. (2024) emphasize that the perception of creaminess is one of the main determinants of acceptance in plant‐based products, while watery or grainy textures tend to reduce consumer liking.

Another important aspect is the interaction between intrinsic product characteristics and consumer attitudes. Della Fontana et al. (2025) found that a soy dessert was more acceptable than a plant‐based dessert with a more intense flavor and rougher texture, with this response also influenced by attitudes toward reducing meat consumption. In the present study, the greater acceptance of dairy versions may also reflect a combination of a more familiar sensory profile and a cultural preference for dairy products among Brazilian children.

Although the acceptance of the SSPE version was lower than that of the CM version, the rate obtained (∼50%) indicates that specific formulation adjustments, such as improving creaminess, masking plant‐based notes, and balancing sweetness, can significantly increase the acceptance index. Thus, there is room for optimizing the plant‐based alternative while maintaining iron intake and physicochemical stability, expanding the offering of non‐dairy options in school feeding programs.

From a nutritional perspective, one 90 g portion adopted in this study corresponds to the standard serving size recommended by the Brazilian national school feeding programme for desserts (BRASIL 2014). Each serving provides approximately 5 mg of total iron, corresponding to 30–35% of the dietary reference intake (DRI) for children aged 4 to 10 years (ANVISA 1998; WHO 2025). When consumed two to three times per week, a frequency consistent with school meal rotation schedules, the fortified dessert shows potential as an effective fortification vehicle within institutional feeding programs.

## 4 Conclusion

Surprisingly, iron absorption inhibitors present in milk, such as Ca^2^⁺ and caseins, did not impair the bioavailability of the mineral, as demonstrated by the comparison between formulations made with CM and SSPE. Both desserts fortified with MDFP promoted the recovery of iron nutritional status in previously depleted rats, reaching approximately 70% of the relative bioavailability of FeSO_4_, taken as a reference. Gene expression analyses of DcytB, DMT‐1, ferroportin, and hephaestin revealed that the use of an extruded rice grain matrix significantly increased iron bioavailability compared to pure MDFP. Furthermore, the expression of these proteins, directly related to iron metabolism, was more pronounced in animals fed diets with lower bioavailability, supporting the hypothesis that iron deficiency stimulates compensatory absorption mechanisms.

In addition to the results obtained in animal models, we sought to verify the potential practical application of the formulations through sensory acceptance tests with children. In this evaluation, all formulations were rated as “good acceptance,” with greater preference for the CM‐based versions, possibly due to their more familiar sensory profile and higher lipid content. Taken together, these results demonstrate that the inclusion of MDFP in the extruded rice matrix constitutes an effective fortification strategy for rice‐based desserts, presenting not only high bioavailability but also good acceptance by children, thus representing an effective tool for the development of food products aimed at mitigating iron deficiency, either for industrial purposes or in public policies to combat children malnutrition.

## Nomenclature

AIN‐93G: Diet for rats in growth, pregnancy and lactation
CM: Cow milk
DcytB: Protein duodenal cytochrome B
*DMT‐1*
: The divalent metal transporter 1
DRI: Dietary Reference Intake
*FER*
: Feed efficiency ratio
FS+AIN‐93G: Diet with ferrous sulfate
*GAPDH*
: Glyceraldehyde 3‐phosphate dehydrogenase
*HCP‐1*
: Heme carrier protein 1
*HRE*
: Hemoglobin Regeneration Efficiency
MDFP+AIN‐93G: Diet with micronized ferric pyrophosphate
MDFP+R+CM: Fortified dessert with rice + cow's milk
MDFP+R+SSPE: Fortified dessert with Rice + SSPE (soluble soy protein extract)
*RBV‐HRE*
: Relative biological value of HRE
RT‐qPCR: Reaction Technique of real‐time Polymerase Chain
SSPE: Soluble soy protein extract

## Author Contributions

**Danielle Cristine Mota Ferreira**: writing – review and editing, writing – original draft, investigation, conceptualization. **Thomás Valente de Oliveira**: investigation, writing – original draft, writing – review and editing, conceptualization. **Maria Eliza Castro Moreira**: writing – review and editing, methodology, validation. **Mônica Ribeiro Pirozi**: methodology, validation, writing – review and editing. **Hércia Stampini Duarte Martino**: methodology, visualization, writing – review and editing. **Eduardo Basílio de Oliveira**: conceptualization, funding acquisition, writing – review and editing, validation, project administration, supervision, resources, data curation.

## Conflicts of Interest

The authors declare no conflicts of interest.
